# Efficacy and safety of dual long-acting injectable antipsychotics in a patient with treatment-resistant schizophrenia: a case report

**DOI:** 10.1192/j.eurpsy.2025.2262

**Published:** 2025-08-26

**Authors:** P. Russo, P. Catapano, S. Cipolla, A. F. Bonamico, F. Perris, F. Catapano

**Affiliations:** 1 Department of Psychiatry, University of Campania “Luigi Vanvitelli”, Naples, Italy

## Abstract

**Introduction:**

Schizophrenia is often characterized by chronic progression and frequent relapses. However, recent evidence suggests that many patients can achieve symptoms remission and functional recovery through integrated pharmacological and psychosocial interventions. Despite treatment advances, up to 34% of patients are resistant to standard antipsychotic treatments, with clozapine being the gold standard for treatment-resistant schizophrenia (TRS). However, up to 60% of TRS patients may not respond adequately to clozapine due to its adverse effects and lack of compliance. Recently, the use of two long-acting injectable (LAI) antipsychotics has emerged as a potential strategy for managing TRS, particularly in patients with poor adherence to oral therapies.

**Objectives:**

The objective is to provide clinical insights into this novel pharmacological approach consisting in the administration of two LAI antipsychotics in patients with treatment resistant schizophrenia.

**Methods:**

This case report describes the clinical management of a 62-year-old woman with a 20-year history of treatment-resistant paranoid schizophrenia, characterized by multiple hospitalizations, delusions, auditory hallucinations, disorganized behavior, and non-adherence to oral medications.

**Results:**

Due to the patient’s poor response to prior treatments, including clozapine, a novel therapeutic strategy was adopted during her hospitalization in March 2023. Two LAI antipsychotics, haloperidol decanoate (100 mg/28 days) and aripiprazole (400 mg/28 days), were administered alternately to optimize symptom control while minimizing adverse effects. Over 8 weeks, the patient demonstrated significant improvements in psychotic symptoms, mood, and functioning. Importantly, adherence to treatment improved.

**Conclusions:**

This case highlights the potential efficacy and safety of combining two LAI antipsychotics in TRS patients, particularly those with poor adherence to oral therapies. The alternating administration of haloperidol and aripiprazole may exploit the synergistic effects of first- and second-generation antipsychotics, offering a promising alternative for managing complex cases of schizophrenia. Further studies are needed to confirm these findings and establish guidelines for dual LAI use in TRS patients.

**Disclosure of Interest:**

None Declared

